# The relationship between childcare and adiposity, body mass and obesity-related risk factors: protocol for a systematic review of longitudinal studies

**DOI:** 10.1186/s13643-016-0312-7

**Published:** 2016-08-17

**Authors:** Silvia Costa, Jean Adams, Veronica Phillips, Sara E Benjamin Neelon

**Affiliations:** 1 UKCRC Centre for Diet and Activity Research, Medical Research Council Epidemiology Unit, School of Clinical Medicine, University of Cambridge, Box 285 Institute of Metabolic Science, Cambridge Biomedical Campus, Cambridge, CB2 0QQ UK; 2 Medical Library, School of Clinical Medicine, University of Cambridge, Box 111, Cambridge Biomedical Campus, Cambridge, CB2 0SP UK; 3 Johns Hopkins Bloomberg School of Public Health, Johns Hopkins University, 624 N Broadway, Baltimore, MD 21205 USA

**Keywords:** Adiposity, Body mass, Childcare, Daycare, Diet, Obesity, Physical activity, Sedentary behaviour, Sleep, Stress

## Abstract

**Background:**

The rising prevalence of obesity, particularly in childhood, is a global public health emergency. There is some evidence that exposure to non-parental childcare before age 6 years is associated with subsequent development of obesity and obesity-related behaviours such as physical activity, sedentary behaviour, sleep, diet and stress, although these findings are inconsistent. It is possible that the relationship between early childcare and later obesity and obesity-related behaviours depends on characteristics of childcare exposure such as type (i.e. informal versus formal care), duration (i.e. number of years spent in childcare), intensity (e.g. number of hours per week) and timing (i.e. age of onset of childcare) of care received. The relationship may also be moderated by socio-demographic characteristics of children and their families. We will conduct a systematic review exploring longitudinal associations between childcare (type, duration, intensity and timing) and measures of adiposity and body mass, physical activity, sedentary behaviour, sleep, diet and stress. We will also assess whether these relationships vary by socio-demographic factors.

**Methods:**

We will include studies that explore longitudinal associations between childcare attendance in children aged <6 years not in primary school at first assessment and body weight, adiposity, physical activity, diet, sleep and stress. We will limit studies to those involving middle- and high-income countries. Two independent reviewers will screen search results in two stages: (1) title and abstract and (2) and full text. One reviewer will extract relevant data and a second will verify this information. We will assess risk of bias of included studies using an adaption of the United States Department of Agriculture National Evidence Library Bias Assessment Tool. We will tabulate and summarise results narratively. We may conduct meta-analysis if at least five studies report comparable data.

**Discussion:**

To our knowledge, this will be the first systematic review to summarise the existing evidence on longitudinal associations between childcare and adiposity, body mass and obesity-related risk factors. The results will be of relevance to other researchers, childcare practitioners and policy makers.

**Systematic review registration:**

PROSPERO CRD42015027233

**Electronic supplementary material:**

The online version of this article (doi:10.1186/s13643-016-0312-7) contains supplementary material, which is available to authorized users.

## Background

High rates of childhood obesity are a worldwide concern [1]. Globally in 2010, approximately 6.7 % (43 million) of preschool-aged children were overweight or obese, and this prevalence is estimated to rise to 9.1 % by 2020 if current trends are maintained [2]. Obesity in childhood is associated with increased risk of both obesity in adulthood and a range of other conditions including low self-esteem, high blood pressure, insulin resistance, coronary heart disease and stroke [3–8].

The early years (<6 years of age) have been repeatedly highlighted as a critical period for the development and prevention of obesity [6, 9–11]. They are also an important period for the establishment of healthy habits, including healthy physical activity, dietary patterns and sleeping patterns [12–14]—all of which can help protect against the development of obesity. Several individual (e.g. sex, dietary and activity behaviours), inter-personal (e.g. peer/sibling interactions, parent feeding practices) and environmental factors (e.g. neighbourhood safety, childcare) influence the development of childhood obesity [15]. As environmental factors affect large numbers of children, they represent potential targets for obesity prevention [16].

An increasing number of children now attend out-of-home childcare prior to 6 years of age, and many spend large proportions of their week days in such care [17, 18]. A recent report by the United Nations Children’s Emergency Fund shows that roughly 80 % of 3–6 year olds and 25 % of 0–3 year olds in developed countries spend time in some form of childcare [17]. As a result, childcare settings have been targeted for intervention efforts to prevent obesity [13, 19–21].

A growing body of research suggests a positive association between childcare attendance and increased adiposity and risk of obesity in children [22–29]. Geoffroy et al. found that Canadian children who attended centre-based childcare or were cared for by a relative between 1.5–4 years of age had higher odds of being overweight or obese at 4–10 years of age than those receiving parental care [22]. Similarly, Lin et al. found that informal childcare (e.g. by grandparents or domestic helpers) at ages 3 and 5 years was associated with increased risk for overweight and higher body mass index *z*-score at age 11, in comparison to those cared for by parents [27].

However, the relationship between non-parental childcare and future obesity in children is not consistent and may partly depend on specific aspects of the childcare received. These aspects include the type (i.e. informal versus formal care), duration (i.e. number of years spent in childcare), intensity (e.g. number of hours per week) and timing (i.e. age of onset of childcare) of care received. Additionally, relationships between non-parental childcare and future risk of obesity may vary according to different socio-demographic characteristics, such as child’s ethnicity and family’s socioeconomic position [30–32]. For example, a study by Zahir et al. reported no association between attendance, age of entry and number of hours spent in non-parental childcare and risk of obesity at 4 years of age in a sample of Latino children [30]. Maher et al. found that Latino children in informal childcare or those who attended Head Start had lower risk of obesity at 4 years than those in parental care [31]. Conversely, non-Latino children in informal childcare had an increased risk of obesity [31]. A more recent study found that care by a non-relative was associated with increased body mass index percentile between 2–3 and 6–7 years for boys of all income levels, but in girls this association was only found for those in low-income households [32].

Furthermore, the pathways through which childcare experiences might affect obesity are poorly understood [33–35]. Different types and characteristics of childcare settings may have different influences on development of obesity-related risk factors, such as physical activity, sedentary behaviour, sleep, diet and stress [19, 36–43]. A recent systematic review identified some childcare staff behaviours (e.g. providing portable play equipment and playing with children) associated with increased physical activity in children in cross-sectional studies [44]. However, this was not seen across all studies investigating these behaviours, and there was a lack of evidence from longitudinal studies. Additionally, whilst some studies reported increased energy intake at childcare (e.g. average lunch intake in US 15–24 month olds: 281 kcal at home versus 332 kcal at childcare) [45], others have reported no difference in energy intake at home versus at childcare [29]. However, given evidence of associations between physical activity, sedentary behaviour, sleep, diet and stress with increased adiposity in early childhood [34, 46–50], we hypothesise that these risk factors are possible pathways through which childcare may influence the development of obesity. To our knowledge, no previous systematic reviews have examined the associations between childcare and adiposity, obesity and obesity-related risk factors.

### Objectives

The objectives of this systematic review are to answer the following research questions:

1. Is childcare (type, duration, intensity and timing) longitudinally associated with measures of adiposity and body mass?
2. Is childcare (type, duration, intensity and timing) longitudinally associated with (a) physical activity, (b) sedentary behaviour, (c) sleep, (d) diet or (e) stress?
3. Do the answers to questions 1 and 2 vary by socio-demographic factors (e.g. family income, parental education, child race/ethnicity)?

## Methods

We will conduct a systematic review of studies exploring the longitudinal relationship between childcare, adiposity or body mass and obesity-related outcomes. The review has been registered on the PROSPERO database (registration number CRD42015027233). Thus far, we have conducted preliminary but not definitive searches. This protocol is reported according to the Preferred Reporting Items for Systematic Review and Meta-Analysis Protocols (PRISMA-P) guidance [see Additional file 1] [51].

### Eligibility criteria

We will select studies for inclusion in the systematic review according to the following criteria.

#### Participants

We will include studies of children aged <6 years and not in primary school at first assessment. Restricting the review to this age range and to children not in primary school allows us to focus on specifically on childcare rather than care provided in school settings and care provided around formal school hours. Formal schooling starts by 6 years of age in the majority of countries.

We will also restrict studies to those investigating children living in middle- and high-income countries. We use the World Bank’s definition of low-income countries as those with a gross national income per capita of less than US$1045 [52]. We will exclude studies from low-income countries due to the differing economic, social and health environments in higher versus lower income settings, including investments in and access to health and education [53, 54], and leading causes of death [55]. These factors are likely to influence use of childcare.

#### Study design

The review will include observational longitudinal studies, including case-control, prospective and retrospective studies. We will exclude other study designs. Restricting the review to observational designs allows us to explore associations between childcare and the outcomes of interest as they exist in the general population and in real-life settings, rather than in experimental settings. Restricting the review to longitudinal studies allows us to examine associations between childcare and adiposity, body mass and obesity-related risk factors over time; longitudinal studies provide a stronger indicator of causal relationships than cross-sectional data [56]. However, we recognise that causality cannot be confirmed with longitudinal data.

#### Exposure

The exposure of interest is non-parental childcare use (overall and by type). If data are available, we will look at different characteristics of childcare such as timing of attendance (i.e. age when care started and stopped), intensity (i.e. full- or part-time), duration (i.e. years of childcare) and types of childcare (i.e. formal or informal; private or public). If applicable (e.g. in case-control studies), the main comparator accepted for inclusion will be parental care. Thus, studies will need to have variation in the exposure.

### Outcomes

#### Primary outcome

The primary outcome of interest is adiposity or body mass, directly measured (e.g. by researchers, childcare staff or parents) at a time point subsequent to recording of childcare use. Examples of accepted measures include weight, body mass index, waist or hip circumference, waist-to-hip ratio, skinfold thickness, fat mass and overweight or obesity status. We will exclude studies using self-reported measures of adiposity.

#### Secondary outcomes

We will include five secondary outcomes variables: physical activity, sedentary behaviour, sleep, diet and stress. As mentioned, these variables were chosen because previous research shows associations between each variable and both childcare and obesity. These variables are hypothesised pathways through which childcare may influence adiposity, body mass or risk of obesity. We will include studies using objectively assessed or proxy/self-reported measures of physical activity, sedentary behaviour, sleep, diet or stress. We will include studies assessing any of these five secondary variables, independently of whether or not they also report on the association between childcare and the primary outcome.

Examples of measures for each of the secondary outcomes include parent-completed physical activity questionnaire or accelerometer-measured physical activity and/or sedentary behaviour; parent-completed sleep diary or accelerometer-measured sleep; parent-completed food frequency questionnaires or directly observed diet behaviours; parent-completed stress questionnaire or salivary cortisol.

### Search methods

We will search the following electronic bibliographic databases for studies that meet the inclusion criteria: MEDLINE, EMBASE, PsycINFO, Web of Science, Scopus, SPORTDiscus with Full Text, Applied Social Sciences Index and Abstracts (ASSIA) and the Scientific Electronic Library Online (SciELO). We will not restrict studies based on publication date or language. We will screen the reference list of included studies and search for articles citing included studies using the science and social science citation indices. We will re-run searches just before the final analyses to identify any recent studies that meet the inclusion criteria for this review.

We developed the search strategy based on the key themes of relevance to this review (i.e. childcare, adiposity and body mass, physical activity, sedentary behaviour, sleep, diet and stress). It was also informed by search strategies of relevant previous systematic reviews [57, 58]. The search strategy was reviewed and will be run by an experienced university librarian, with results provided to the reviewers in an EndNote® database. An example of the final search strategy used for the MEDLINE database is provided [see Additional file 2].

### Selection of studies

One reviewer (SC) will screen the EndNote® database for duplicate records for exclusion. Two reviewers will then independently screen the studies identified by the searches following a two-phase procedure. We have piloted the procedures for both screening phases using a sample of relevant articles, revised them and agreed on the final format according to the results of this work. For phase 1, title and abstract of articles identified by the searches will be screened against the following criteria:

1. Age of children at baseline: <6 years? (yes, no or not clear)
2. Location of study: high- or middle-income country? (yes, no or not clear)
3. Study design: observational longitudinal design? (yes, no or not clear)
4. Exposure: was childcare investigated as an exposure? (yes, no or not clear)
5. Outcomes: were target outcomes investigated in the study? (yes, no or not clear)

We will retrieve full texts of all studies identified by either screener as potentially eligible (i.e. answers to all five screening questions are “yes” or “not clear”). In phase 2, we will screen full texts against the following criteria:

1. Age of children at baseline: <6 years? (yes or no)
2. Location of study: high- or middle-income country? (yes or no)
3. Study design: observational longitudinal design? (yes or no)
4. Exposure: was childcare investigated as an exposure? (yes or no)
  4.1 Is there a variation in the exposure? (yes or no)
5. Outcomes: were target outcomes investigated in the study? (yes or no for each of the potential outcomes)
  5.1. For adiposity/body mass outcome: was this directly measured? (yes or no)

We will include studies meeting all the above inclusion criteria (i.e. “yes” to all 5 questions) in the review. We will resolve disagreements between reviewers regarding the eligibility of particular studies through a collective discussion including the third reviewer.

### Data extraction and management

We will use a standardised and pre-piloted form to extract data from the included studies for assessment of study quality and evidence synthesis. Extracted information will include study ID; PubMed ID; author; year; country; study setting; study population and baseline participant demographics and characteristics; details of the exposure; duration of follow-up; measurement methods (e.g. subjective or objective) and characteristics of exposure and outcome variables (e.g. type and duration of childcare); study design; recruitment and study completion rates; outcomes and times of measurement; statistical analysis used to assess associations between exposure and outcome variables; covariates included in analyses; amount of missing data; results (e.g. odds or hazards ratios, beta coefficients of relationships between childcare and any of the outcomes, including significant and non-significant associations); and information needed for assessment of the risk of bias.

The first author will extract these data and a second author will independently check the extracted information. We will request missing data from study authors. We will record extracted information in an electronic database, which will be archived and available for access by all reviewers in a shared electronic folder.

### Quality assessment

We will assess risk of bias of individual studies using an adaptation of the United States Department of Agriculture Nutrition Evidence Library Bias Assessment Tool [59]. This tool assesses risk of selection, performance, detection and attrition bias. For observational studies, responses to each of the 12 questions are scored 0–2, giving a possible range of scores of 0–24, with lower scores indicating lower risk of bias. We will describe the range of scores present for each question and for the tool overall for all included studies. Two reviewers will independently assess all included studies for risk of bias, and any disagreements will be discussed and resolved with the third reviewer.

### Evidence synthesis

We will tabulate key information (e.g. sample characteristics, exposure and outcome characteristics) grouped by outcome and exposure variables and perform a narrative synthesis. In this narrative synthesis, we will attempt to explore differences between the following: (1) the type, duration, intensity and timing of childcare; (2) each of the outcome variables; (3) variants of secondary outcomes (e.g. subjective and objective measures of physical activity or sedentary behaviour); (4) high- and middle-income countries; and (5) infants, toddlers and pre-schoolers. We will also explore the possibility of reporting results for different socio-demographic sub-groups (e.g. by ethnicity).

We will conduct a separate meta-analysis for each of the primary and secondary outcomes if there are at least five studies with comparable exposure and outcome variables. In these cases, random or fixed effects meta-analyses will be conducted as appropriate. Where adjusted and unadjusted results are presented in primary papers, we will include the most adjusted results available in meta-analyses and results from different study designs will be pooled. Heterogeneity will be explored using the *I*^2^ statistic. Where *I*^2^ values are 50 % or more, we will conduct sub-group analyses to explore this. Planned sub-group analyses (for each meta-analysis) will focus on different measures of each outcome and exposure as described above and on different study designs. Funnel plots will be used to explore publication bias. Sensitivity analysis will explore the effect of excluding studies that appear to be statistical outliers. Finally, we will prepare a “summary of findings” table as described in the Cochrane Handbook and use the GRADE approach for describing the quality of relevant evidence.

### Amendments to protocol

Any substantive amendments to this protocol will be registered with PROSPERO as they occur and documented in the final publication.

### Dissemination

We will publish review results in an international peer-reviewed journal and will report results according to the Preferred Reporting Items for Systematic Reviews and Meta-Analyses (PRISMA) statement. Additionally, we will publish a lay summary on the website of the Medical Research Council Epidemiology Unit. We will also disseminate results to the research community and relevant key stakeholders (e.g. policy makers and national associations of childcare providers) through presentations at relevant academic and non-academic meetings and via social media (e.g. Twitter). If findings are found to be interesting to the wider public, we will disseminate them via mass media.

## Discussion

To our knowledge, this will be the first systematic review to summarise the existing evidence on longitudinal associations between childcare and adiposity, body mass and obesity-related risk factors. An increasing number of children worldwide are exposed to non-parental childcare from a very early age and for long periods of the day [17, 18]. Some governments support early childcare use in order to encourage parents (particularly those from lower income families) back into work and so provide the best start for their children [60]. However, if non-parental childcare has negative impacts on children’s lifetime risk of obesity and obesity-related risk factors, these policies may be at odds with other policies and actions aiming to halt and reverse current trends in childhood obesity.

Results from this review will help to identify characteristics of non-parental childcare associated with subsequent increased risk of obesity and obesity-related risk factors. This information will be useful for researchers, childcare practitioners and policy makers to guide the development of interventions and strategies to prevent the development of obesity and establish healthy habits from an early age.

## Abbreviations

ASSIA, Applied Social Sciences Index and Abstracts; PRISMA, Preferred Reporting Items for Systematic Review and Meta-Analysis; SciELO, Scientific Electronic Library Online

## Additional files

Additional file 1: PRISMA-P checklist. (DOCX 41 kb) Additional file 2: Search strategy. (DOCX 27 kb)
