# Supplementary material for: The relationship between childcare and adiposity, body mass and obesity-related risk factors: protocol for a systematic review of longitudinal studies
Source: Syst Rev. 2016 Aug 17;5:141. doi: 10.1186/s13643-016-0312-7 (PMC4989341; doi:10.1186/s13643-016-0312-7)
Supplement: Additional file 2: — Search strategy. (DOCX 27 kb) [file 13643_2016_312_MOESM2_ESM.docx]

Medline via Ovid:

1. childcare.ti,ab.

2. "child care".ti,ab.

3. "family care".ti,ab.

4. "care cent*".ti,ab.

5. preschool*.ti,ab.

6. "pre school*".ti,ab.

7. nurser*.ti,ab.

8. kindergarten*.ti,ab.

9. creche*.ti,ab.

10. childmind*.ti,ab.

11. nann*.ti,ab.

12. "au pair*".ti,ab.

13. "friend care".ti,ab.

14. "neighbor care".ti,ab.

15. "neighbour care".ti,ab.

16. "kith care".ti,ab.

17. "kin care".ti,ab.

18. "play school".ti,ab.

19. "play group".ti,ab.

20. "early years".ti,ab.

21. "age integrated".ti,ab.

22. "grandparent care".ti,ab.

23. or/1-22

24. "body composition".ti,ab.

25. fat*.ti,ab.

26. adipos*.ti,ab.

27. lean*.ti,ab.

28. waist.ti,ab.

29. abdominal.ti,ab.

30. hip.ti,ab.

31. weight.ti,ab.

32. BMI.ti,ab.

33. quetelet.ti,ab.

34. "ponderal index".ti,ab.

35. obes*.ti,ab.

36. overweight.ti,ab.

37. underweight.ti,ab.

38. thin*.ti,ab.

39. "skin fold thickness".ti,ab.

40. DEXA.ti,ab.

41. DXA.ti,ab.

42. "dual energy x-ray absorptiometry".ti,ab.

43. impedance.ti,ab.

44. anthropometer*.ti,ab.

45. or/24-44

46. activ*.ti,ab.

47. play*.ti,ab.

48. exercise*.ti,ab.

49. "motor skill*".ti,ab.

50. walk*.ti,ab.

51. "motor development".ti,ab.

52. step*.ti,ab.

53. sport*.ti,ab.

54. fitness.ti,ab.

55. "gross motor".ti,ab.

56. game*.ti,ab.

57. or/46-56

58. sedentar*.ti,ab.

59. inactiv*.ti,ab.

60. television.ti,ab.

61. tv.ti,ab.

62. screen*.ti,ab.

63. computer.ti,ab.

64. "video game*".ti,ab.

65. "electronic game*".ti,ab.

66. DVD.ti,ab.

67. internet.ti,ab.

68. or/58-67

69. diet*.ti,ab.

70. nutrition*.ti,ab.

71. eat*.ti,ab.

72. food*.ti,ab.

73. fruit*.ti,ab.

74. vegetable*.ti,ab.

75. water.ti,ab.

76. juice*.ti,ab.

77. sugar*.ti,ab.

78. fizzy.ti,ab.

79. drink*.ti,ab.

80. meal*.ti,ab.

81. "take away".ti,ab.

82. "energy dense".ti,ab.

83. "high fat".ti,ab.

84. "low fat".ti,ab.

85. "nutrient poor".ti,ab.

86. "empty calor*".ti,ab.

87. "portion size*".ti,ab.

88. confectionary.ti,ab.

89. sweet*.ti,ab.

90. dessert*.ti,ab.

91. chocolate*.ti,ab.

92. cake*.ti,ab.

93. biscuit*.ti,ab.

94. chip*.ti,ab.

95. crisp*.ti,ab.

96. "french fries".ti,ab.

97. snack*.ti,ab.

98. meat*.ti,ab.

99. cereal*.ti,ab.

100. breakfast*.ti,ab.

101. lunch*.ti,ab.

102. dinner*.ti,ab.

103. fried.ti,ab.

104. burger*.ti,ab.

105. "breastfeed*".ti,ab.

106. "breast milk".ti,ab.

107. "bottle feed*".ti,ab.

108. formula.ti,ab.

109. candy.ti,ab.

110. beverage*.ti,ab.

111. savory.ti,ab.

112. savoury.ti,ab.

113. overeat*.ti,ab.

114. or/69-113

115. sleep*.ti,ab.

116. rest*.ti,ab.

117. nap*.ti,ab.

118. or/115-117

119. stress*.ti,ab.

120. cortisol.ti,ab.

121. HPA.ti,ab.

122. "hypothalamic pituitary adrenal".ti,ab.

123. catecholamine*.ti,ab.

124. ACTH.ti,ab.

125. or/119-124

126. Epidemiologic studies/

127. exp case control studies/

128. exp cohort studies/

129. Case control.tw.

130. (cohort adj (study or studies)).tw.

131. Cohort analy$.tw.

132. (Follow up adj (study or studies)).tw.

133. (observational adj (study or studies)).tw.

134. Longitudinal.tw.

135. Retrospective.tw.

136. or/126-135

137. 23 and 45 and 136

138. 23 and 57 and 136

139. 23 and 68 and 136

140. 23 and 118 and 136

141. 23 and 125 and 136

142. 23 and 114 and 136

143. limit 137 to humans

144. limit 138 to humans

145. limit 139 to humans

146. limit 140 to humans

147. limit 141 to humans

148. limit 142 to humans

Embase via Ovid:

1. childcare.ti,ab.

2. "child care".ti,ab.

3. "family care".ti,ab.

4. "care cent*".ti,ab.

5. preschool*.ti,ab.

6. "pre school*".ti,ab.

7. nurser*.ti,ab.

8. kindergarten*.ti,ab.

9. creche*.ti,ab.

10. childmind*.ti,ab.

11. nann*.ti,ab.

12. "au pair*".ti,ab.

13. "friend care".ti,ab.

14. "neighbor care".ti,ab.

15. "neighbour care".ti,ab.

16. "kith care".ti,ab.

17. "kin care".ti,ab.

18. "play school".ti,ab.

19. "play group".ti,ab.

20. "early years".ti,ab.

21. "age integrated".ti,ab.

22. "grandparent care".ti,ab.

23. or/1-22

24. "body composition".ti,ab.

25. fat*.ti,ab.

26. adipos*.ti,ab.

27. lean*.ti,ab.

28. waist.ti,ab.

29. abdominal.ti,ab.

30. hip.ti,ab.

31. weight.ti,ab.

32. BMI.ti,ab.

33. quetelet.ti,ab.

34. "ponderal index".ti,ab.

35. obes*.ti,ab.

36. overweight.ti,ab.

37. underweight.ti,ab.

38. thin*.ti,ab.

39. "skin fold thickness".ti,ab.

40. DEXA.ti,ab.

41. DXA.ti,ab.

42. "dual energy x-ray absorptiometry".ti,ab.

43. impedance.ti,ab.

44. anthropometer*.ti,ab.

45. or/24-44

46. activ*.ti,ab.

47. play*.ti,ab.

48. exercise*.ti,ab.

49. "motor skill*".ti,ab.

50. "motor development".ti,ab.

51. walk*.ti,ab.

52. step*.ti,ab.

53. sport*.ti,ab.

54. fitness.ti,ab.

55. "gross motor".ti,ab.

56. game*.ti,ab.

57. or/46-56

58. sedentar*.ti,ab.

59. inactiv*.ti,ab.

60. television.ti,ab.

61. tv.ti,ab.

62. screen*.ti,ab.

63. computer.ti,ab.

64. "video game*".ti,ab.

65. "electronic game*".ti,ab.

66. DVD.ti,ab.

67. sit*.ti,ab.

68. or/58-67

69. diet*.ti,ab.

70. nutrition*.ti,ab.

71. eat*.ti,ab.

72. food*.ti,ab.

73. fruit*.ti,ab.

74. vegetable*.ti,ab.

75. water.ti,ab.

76. juice*.ti,ab.

77. sugar*.ti,ab.

78. fizzy.ti,ab.

79. drink*.ti,ab.

80. meal*.ti,ab.

81. "take away".ti,ab.

82. "energy dense".ti,ab.

83. "high fat".ti,ab.

84. "low fat".ti,ab.

85. "nutrient poor".ti,ab.

86. "empty calor*".ti,ab.

87. "portion size*".ti,ab.

88. confectionary.ti,ab.

89. sweet*.ti,ab.

90. dessert*.ti,ab.

91. chocolate*.ti,ab.

92. cake*.ti,ab.

93. biscuit*.ti,ab.

94. chip*.ti,ab.

95. crisp*.ti,ab.

96. "french fries".ti,ab.

97. snack*.ti,ab.

98. meat*.ti,ab.

99. cereal*.ti,ab.

100. breakfast*.ti,ab.

101. lunch*.ti,ab.

102. dinner*.ti,ab.

103. fried.ti,ab.

104. burger*.ti,ab.

105. "breastfeed*".ti,ab.

106. "breast milk".ti,ab.

107. "bottle feed*".ti,ab.

108. formula.ti,ab.

109. candy.ti,ab.

110. beverage*.ti,ab.

111. savoury.ti,ab.

112. savory.ti,ab.

113. overeat*.ti,ab.

114. or/69-113

115. sleep*.ti,ab.

116. rest*.ti,ab.

117. nap*.ti,ab.

118. or/115-117

119. stress*.ti,ab.

120. cortisol.ti,ab.

121. HPA.ti,ab.

122. "hypothalamic pituitary adrenal".ti,ab.

123. catecholamine*.ti,ab.

124. ACTH.ti,ab.

125. or/119-124

126. Clinical study/

127. "Case control study".mp. [mp=title, abstract, heading word, drug trade name, original title, device manufacturer, drug manufacturer, device trade name, keyword]

128. Family study/

129. Longitudinal study/

130. Retrospective study/

131. Prospective study/

132. Randomized controlled trials/

133. 131 not 132

134. Cohort analysis/

135. (Cohort adj (study or studies)).mp.

136. (Case control adj (study or studies)).tw.

137. (follow up adj (study or studies)).tw.

138. (observational adj (study or studies)).tw.

139. (epidemiologic$ adj (study or studies)).tw.

140. (cross sectional adj (study or studies)).tw.

141. or/126-130,133-140

142. 23 and 45 and 141

143. 23 and 57 and 141

144. 23 and 68 and 141

145. 23 and 114 and 141

146. 23 and 118 and 141

147. 23 and 125 and 141

148. limit 142 to human

149. limit 143 to human

150. limit 144 to human

151. limit 145 to human

152. limit 146 to human

153. limit 147 to human
